# The Experiences and Perceptions of Organizational Support Among Chinese Nurses Following Work‐Related Musculoskeletal Disorders: A Qualitative Descriptive Study

**DOI:** 10.1002/nop2.70793

**Published:** 2026-09-07

**Authors:** Kejimu Sunzi, Yihong Lin, Qi Huang, Li Guo, Zhiyun Yang, Fan Li, Wen Zhou, Cheng Lei

**Affiliations:** ^1^ Department of Pediatrics Deyang People's Hospital Deyang China; ^2^ Department Critical Care Medicine The Sixth People's Hospital of Xinjiang Uygur Autonomous Region Urumqi China; ^3^ Department of Gastroenterology The Second Affiliated Hospital of Chongqing Medical University Chongqing China; ^4^ Department of Otorhinolaryngology‐Head and Neck Surgery Chongqing People's Hospital, Chongqing University Chongqing China; ^5^ Department of Nursing The Second Affiliated Hospital of Chongqing Medical University Chongqing China; ^6^ Department of Thoracic Surgery, Sichuan Clinical Research Center for Cancer, Sichuan Cancer Hospital & Institute, Sichuan Cancer Center The Affiliated Cancer Hospital of University of Electronic Science and Technology of China Chengdu China

**Keywords:** Chinese nurses, occupational injuries, organizational support, qualitative research, work‐related musculoskeletal disorders

## Abstract

**Background:**

Work‐related musculoskeletal disorders (WMSDs) are highly prevalent among nurses in China and can affect nurses' health, work ability, professional identity, and retention. Although Organizational Support Theory suggests that perceived organizational support shapes employees' post‐injury attitudes and behaviours, little qualitative evidence explains how Chinese nurses interpret organizational responses after WMSDs, especially in the context of high clinical workloads and staffing pressure.

**Objective(s):**

To explore and describe the experiences and perceptions of organizational support among Chinese nurses following WMSDs.

**Design:**

A qualitative descriptive study.

**Methods:**

Twenty‐four Chinese registered nurses from five tertiary hospitals who had taken sick leave or sought medical care for WMSDs in the past two years were recruited via purposive sampling. Data were collected through semi‐structured, one‐on‐one, in‐depth interviews, with each participant completing one interview between January and June 2025. Data were analysed using Braun and Clarke's six‐stage thematic analysis.

**Results:**

Three core themes emerged: (1) Navigating between ambiguous formal systems and crucial informal support, which revealed nurses' struggles between indifferent official procedures and a reliance on personal networks; (2) The chasm between perceived value and organizational actions, which highlighted the discrepancy between the organization's professed ‘care’ and the transactional, punitive measures nurses actually experienced; and (3) Long‐term ramifications of inadequate support on professional identity and career trajectory, which illustrated how poor support experiences eroded organizational loyalty, fostered turnover intentions, and ultimately destabilized their professional foundation.

**Conclusions:**

Organizational support for Chinese nurses following WMSDs was fragmented, reactive, and insufficient, with formal procedures often failing to match the responsiveness of informal peer and manager support. These findings emphasize the need for nursing leaders and hospital administrators to develop structured return‐to‐work pathways, protect injured nurses from financial and career penalties, and strengthen ergonomic prevention as part of workforce retention strategy.

**Patient or Public Contribution:**

No patients or members of the public were involved in the design or conduct of this study. The research focused on the professional experiences of registered nurses, who contributed as participants through one‐on‐one interviews and assisted in the validation of findings through member checking.

**Implications for the Profession and/or Patient Care:**

This study clarifies how nurses' post‐injury experiences are shaped by the gap between informal relational support and formal organizational systems. For nursing practice and management, the findings suggest that reliance on informal peer support is unsustainable and may intensify guilt, presenteeism, and risk to patient care. Standardized phased return‐to‐work programmes, protection from punitive performance‐pay consequences, nurse‐manager training, and investment in ergonomic equipment are practical steps for maintaining a healthy and stable nursing workforce.

**Impact:**

*What problem did the study address?* This study addressed the lack of in‐depth qualitative evidence regarding how Chinese nurses experience and perceive organizational support following WMSDs, particularly within the context of systemic nursing shortages. *What were the main findings?* The study identified a ‘fractured’ support system where formal bureaucratic procedures often fail to recognize individual suffering, forcing nurses to rely on precarious informal networks. This inadequacy severely erodes organizational loyalty, professional identity, and significantly increases turnover intentions. *Where and on whom will the research have an impact?* These findings will impact hospital administrators and nursing leaders by providing a roadmap for reforming occupational health policies. It also serves as a critical reference for health policymakers to develop national guidelines for managing occupational injuries in healthcare settings.

## 1 Introduction

Nursing is physically demanding, and work‐related musculoskeletal disorders (WMSDs) remain one of the most persistent occupational health problems affecting nurses globally (Clari et al. 2021; Gorce and Jacquier‐Bret 2025; Kgakge et al. 2024; Zaitoon et al. 2024). In China, the issue is especially salient: a systematic review and meta‐analysis reported an overall WMSD prevalence of 79% among Chinese clinical nurses (Wang et al. 2024). Heavy workloads, frequent night shifts, manual patient handling, prolonged awkward postures, and repetitive work in confined clinical spaces all increase the risk of cumulative musculoskeletal injury (Wang et al. 2024; Ren et al. 2025; Yin et al. 2025; Sisala Mohammed et al. 2024; Nemera et al. 2024). These risks are intensified by nursing shortages and imbalanced nurse‐to‐patient ratios, which place sustained physical pressure on front‐line nurses (Wang et al. 2025; Tang et al. 2023).

The consequences of WMSDs extend beyond pain. Nurses with WMSDs may experience reduced work efficiency, presenteeism, anxiety, sleep disturbance, limitations in daily life, and stronger intentions to leave clinical work (Talutis et al. 2025; Luna et al. 2025; Min 2022; Nihei et al. 2022; van der Burg et al. 2020; Zeng et al. 2025; Hou et al. 2025; Soares and Porto 2025; Jung and Moon 2025; He et al. 2025; Abolfotouh et al. 2015; Daher et al. 2025; Ibrahim et al. 2020; Phillips et al. 2025). These outcomes are not only individual health problems; they also threaten workforce stability and patient‐care continuity in settings already affected by staffing constraints.

Organizational Support Theory (OST) provides a useful lens for examining this problem. Its central concept, perceived organizational support, refers to employees' beliefs about whether the organization values their contributions and cares about their well‐being (Rhoades and Eisenberger 2002). When employees perceive strong support, they are more likely to reciprocate through commitment and engagement; when support is perceived as weak, burnout, emotional detachment, and turnover intentions may increase (Giessner et al. 2023; Kazemi et al. 2025; Chen et al. 2024; Eisenberger et al. 2002). For nurses with WMSDs, organizational responses to injury, sick leave, and return to work therefore become critical signals of professional value and institutional care.

Existing studies have documented the prevalence and risk factors of WMSDs among Chinese nurses and have acknowledged the importance of organizational support (Wang et al. 2024; Ren et al. 2025; Yin et al. 2025; Kazemi et al. 2022; Xu et al. 2025; Rigi et al. 2025). However, the research problem remains underdeveloped: little is known about how Chinese nurses experience formal and informal support after a WMSD, how these responses shape their return‐to‐work decisions, and how organizational actions affect their professional identity and retention intentions. This study addresses this gap by exploring and describing Chinese registered nurses' experiences and perceptions of organizational support after WMSDs.

## 2 Methods

### 2.1 Study Design

This study employed a qualitative descriptive design. This design was chosen because the aim was to provide a rich, experience‐near account of nurses' perceptions of organizational support after WMSDs rather than to generate a new theory or test causal relationships. Qualitative description is particularly appropriate in nursing and health services research when findings need to remain close to participants' accounts and be readily translatable into clinical and organizational practice (Kämäräinen et al. 2025; Vaismoradi et al. 2013). The study was reported in accordance with the 32‐item Consolidated Criteria for Reporting Qualitative Research (COREQ) checklist (Tong et al. 2007).

### 2.2 Ethical Considerations

The research protocol was reviewed and approved by Deyang People's Hospital (Approval No. 2025‐04‐043‐K01). Although this study involved non‐invasive interviews with healthcare professionals and was considered low‐risk, the research team strictly adhered to the Declaration of Helsinki throughout the process.

Before each interview, all participants were provided with a detailed Information Sheet explaining the study's purpose, the voluntary nature of participation, and their right to withdraw at any time without any negative impact on their employment or career. Written informed consent was obtained from each participant before audio recording began. To ensure confidentiality, all personal identifiers (e.g., names, specific department units) were removed during transcription, and participants were assigned codes (e.g., N1, N2) for data analysis and reporting.

### 2.3 Research Team and Reflexivity

Interviews were primarily conducted by two members of the research team, both female Master's‐educated nurses with clinical backgrounds and advanced training in qualitative inquiry. To minimize social desirability bias, no prior personal or professional relationships existed between the interviewers and participants. Before each session, researchers identified themselves as independent investigators to establish a neutral yet trusting environment, clarifying that the study was independent of hospital administration.

The research team's shared nursing background facilitated an ‘insider’ perspective, fostering immediate rapport and a nuanced understanding of the clinical context. However, this shared identity also posed the risk of ‘common‐sense assumptions’ where researchers might overlook systemic issues due to over‐familiarity. To ensure analytical objectivity, the team maintained a rigorous reflexive stance through regular meetings and the use of reflexive journals.

A pivotal reflexive moment occurred during the analysis of the ‘guilt’ theme. Initially, the researchers, drawing on their own clinical experiences, interpreted participants' guilt as an expected professional emotional response. However, through reflexive dialogue, we realized this guilt was not merely individual but was structurally intensified by the absence of institutional ‘float pools’ and the resulting burden on colleagues. This insight prompted us to re‐examine the data through the lens of systemic failure rather than individual psychology, significantly deepening the final thematic interpretation.

### 2.4 Participants and Setting

A purposive sampling strategy was used to recruit participants who could provide information‐rich accounts relevant to the research question. Participants were recruited from five large tertiary hospitals in mainland China. These hospitals provided inpatient and emergency care and included high‐WMSD‐risk clinical settings such as intensive care units, emergency departments, operating rooms, internal medicine departments, and surgical departments. Inclusion criteria were: (1) registered nurses working in mainland China; and (2) having experienced at least one WMSD event requiring sick leave or medical intervention within the past two years. To achieve maximum variation in the sample, the research team sought diversity in age, gender, clinical department, education level, and years of work experience.

Participants were recruited through posters in partner hospitals and information shared on social networks. Interested nurses were contacted by researchers via phone or email for a preliminary discussion and detailed explanation of the study. Recruitment ceased when the research team determined that data saturation had been reached, as no new concepts or themes were emerging from new interviews. A total of 27 potential interviewees from five hospitals were contacted. Three declined to participate because of scheduling conflicts, leading to a final sample of 24 nurses. After the 20th interview, the initial coding framework became stable. The final four interviews (N21–N24) provided additional depth to existing themes but yielded no new conceptual categories, confirming data saturation.

All interviews were conducted between January and June 2025. To ensure privacy and comfort, participants chose the interview location, which was typically a quiet private room or an online video call. No one other than the researcher and participant was present during the interviews.

### 2.5 Data Collection

Data were collected using semi‐structured, one‐on‐one, in‐depth interviews. Each participant completed one interview only; no repeat interviews were conducted. An open‐ended interview guide was developed based on a literature review and the study's objectives. The guide covered the circumstances of the WMSD event, the injury reporting process, experiences with sick leave and returning to work, interactions with managers and colleagues, and overall perceptions of organizational care (Supporting Information S1). The guide was pilot‐tested with two nurses and slightly refined based on their feedback. Each interview lasted approximately 30 to 60 min. With participants' consent, all interviews were audio‐recorded. Immediately following each interview, the researcher wrote field notes to document the context, non‐verbal cues, and initial research reflections.

### 2.6 Data Analysis

All audio recordings were transcribed verbatim. The data were systematically analysed using the six‐step thematic analysis approach proposed by Braun and Clarke (Braun and Clarke 2006). The process involved: (1) familiarizing with the data through repeated reading; (2) generating initial codes; (3) searching for potential themes; (4) reviewing and refining themes; (5) defining and naming themes; and (6) writing the final report.

To ensure the credibility of the analysis, two members of the research team independently coded the first five transcripts, then met to discuss and reach a consensus, forming a preliminary codebook. Subsequent transcripts were coded by one researcher, with regular team meetings to discuss emerging codes and themes and resolve discrepancies. All themes were derived inductively from the data. Data management was facilitated by NVivo 12 software. A summary of the main findings was shared with five participants for member checking; all confirmed that the results accurately reflected their experiences. We acknowledge that member checking with five participants represents a limited validation strategy; therefore, it was used as one credibility check alongside independent coding, reflexive journaling, and team discussion rather than as the sole basis for confirming the analysis.

## 3 Results

A total of 24 nurses participated in the study. Their demographic and occupational characteristics are summarized in Table 1. Through an in‐depth analysis of the interview data, three core themes and twelve sub‐themes were identified, comprehensively presenting the nurses' experiences of organizational support following WMSDs.

**TABLE 1:** Summary of participant characteristics (*n* = 24).

Characteristic	Category/statistic	Value
Gender	Female	20 (83.3)
	Male	4 (16.7)
Age (years)	Mean ± SD (range)	34.5 ± 6.8 (25–48)
Years of work	Mean ± SD (range)	12.4 ± 7.0 (3–25)
Education	BSc	22 (91.7)
	MSc	2 (8.3)
Department	ICU	8 (33.3)
	DEM	4 (16.7)
	DIM	5 (20.8)
	DS	3 (12.5)
	OR	4 (16.7)

Abbreviations: BSc, Bachelor of Science in Nursing; DEM, Department of Emergency Medicine; DIM, Department of Internal Medicine; DS, Department of Surgery; ICU, Intensive Care Unit; MSc, Master of Science in Nursing; OR, Operating Room.

### 3.1 Theme 1: Navigating Between Ambiguous Formal Systems and Crucial Informal Support

This theme depicts a dual‐track support system that nurses encountered post‐injury. On one track was the hospital's official, procedural, yet often distant and opaque formal system. On the other was the immediate, humanized, but relationship‐dependent informal support from direct supervisors and colleagues. The nurses' entire recovery and return‐to‐work journey was an experience of constantly navigating and struggling between these two disparate forms of support.

#### 3.1.1 The ‘Invisibility’ of Pain in Formal Processes

Participants widely reported that the hospital's injury‐reporting and sick‐leave approval processes were standardized and impersonal. These procedures were designed to document events and file paperwork rather than genuinely understand and respond to individual suffering. The nurses' pain was a subjective experience, and unless there was clear radiological evidence, this ‘invisible’ suffering was often downplayed, questioned, or dismissed as ‘not serious enough’ by the cold, bureaucratic system.

When you go to report a work injury, you just fill out a pile of forms. The people in HR look at you as if to say, ‘Here comes another one.’ They care about your diagnostic certificate, your CT scan, not how much pain you're in or if you can't sleep at night. Your pain, in that process, seems non‐existent. (N2)

When I asked for sick leave, my department understood, but at the hospital level, it felt like a hassle. It's as if you should just keep going as long as you can still walk. Once, my back hurt so much I couldn't stand up straight, and when I went to ask for leave, an administrator asked me, ‘Is it really that serious?’ That moment, I felt so wronged. (N16)

#### 3.1.2 The Nurse Manager: A Critical Fulcrum of the Support System

Across all interviews, the nurse manager was unanimously identified as the ‘key person’ who determined the quality of an injured nurse's subsequent experience. Her attitude and actions directly represented the organization's immediate ‘warmth.’ An empathetic and proactive nurse manager could act as a buffer, shielding the nurse from administrative pressure, flexibly adjusting schedules, and even advocating for their rights.

Our nurse manager is truly wonderful. After I got injured, she immediately sent me home to rest and proactively rearranged my shifts for the next month, letting me do some paperwork. She even spoke to the director of nursing to get me the longest possible recovery time. Without her, I probably would have just endured and kept working. (N7)

Conversely, an indifferent or ineffective manager amplified the organization's neglect, leaving the nurse feeling isolated and helpless.

The nurse manager in my department at the time was overwhelmed herself. I told her my back was failing, and she just said, ‘Everyone's back hurts. Just hang in there, we're short‐staffed.’ It felt like my physical problem was just another hassle for her. At that moment, you completely lose faith in the team. (N4)

#### 3.1.3 Collegial ‘Camaraderie’: A Double‐Edged Sword

Beyond the nurse manager, support from colleagues formed another vital informal network. Co‐workers would proactively take on heavy physical tasks, such as turning and lifting patients. This ‘camaraderie’ provided invaluable support at critical times. However, this support was not without its cost. Over‐reliance on colleagues induced a strong sense of guilt and indebtedness in the injured nurse.

The colleagues in my department are amazing. When they saw me struggling to bend over, they would rush to help turn patients. I was so touched; I could not have lasted a day without them. But after a while, you feel bad yourself. They have their own patients to care for; you cannot trouble others forever, right? (N10)

This psychological burden became a powerful driver for them to return to work quickly, even while still in pain, a phenomenon closely linked to presenteeism. The prevalence of this informal support also highlighted the lack of institutional solutions.

When you're on leave, someone else has to cover your shifts. The department is already short‐staffed, so it's common for one person to do the work of two. You're lying at home, but all you can think about is whether the unit is going crazy and if everyone is talking behind your back. That pressure is worse than the physical pain. So you just think, as soon as I can move, I should get back to work. (N9)

#### 3.1.4 Ambiguous Return‐to‐Work Pathways

Participants universally stated that their hospitals lacked clear, structured, and phased return‐to‐work plans. So‐called ‘light‐duty’ or ‘modified’ positions were often ad‐hoc arrangements, varying from person to person and highly dependent on the nurse manager's discretion and the department's circumstances.

They say you can come back and do some light work, but no one gives you a clear answer on what you'll do or for how long. The nurse manager just arranges things as she sees fit—today you answer phones, tomorrow you do charting. You feel like an ‘outsider,’ detached from normal work, and you feel so insecure. (N18)

This lack of a formal process was filled with uncertainty, making nurses feel their situation was temporary and precarious, which in turn fostered deep anxiety about their future career development.

There is no ‘return‐to‐work process.’ It's just, the doctor says you can go back to work, so you go back. Once you're back, you do what you've always done, maybe just being a bit more careful yourself. No one assesses if your physical condition is truly ready for a full return. You just have to bear it all on your own. (N15)

### 3.2 Theme 2: The Chasm Between Perceived Value and Organizational Actions

This theme directly resonates with the core of OST, profoundly revealing the vast rift between the nurses' perceived self‐worth and the organization's actual behaviours. While hospitals verbally promoted slogans like ‘employees are our greatest asset,’ their actions at the critical moment of a nurse's injury often conveyed the opposite message. This incongruence between words and deeds was a primary source of psychological distress and disillusionment for the nurses.

#### 3.2.1 The Transactional Nature of Support: Care or Cost Control?

Many nurses keenly perceived that the ‘support’ offered by the organization was transactional rather than relational. Measures such as pressuring them to return to work and strictly controlling sick leave days seemed fundamentally motivated by a desire to reduce labor costs and maintain departmental operations, not by genuine concern for employee well‐being.

The HR department and unit leaders call to ‘check on you,’ asking when you can come back to work. They sound polite, but you can feel that they don't care about your back; they care about the vacant position. To them, you're not an injured person but a human resource gap that needs to be filled. (N3)

The entire process focused on how to get the nurse to ‘regain labor capacity’ as quickly as possible, rather than how to ensure their ‘sustainable recovery.’

I feel like whether the hospital treats you well depends on whether you can work. If you can work, you're a treasure; if you can't, they wish you wouldn't take up a spot. It's a very pragmatic and hurtful feeling. (N14)

#### 3.2.2 The ‘Implicit Penalty’ of Injury

Although hospitals had no explicit policies to punish injured employees, nurses universally felt an intangible, underlying ‘penalty.’ They were deeply worried that their injury and sick leave would directly affect their performance evaluations, opportunities for promotion, and especially their year‐end bonuses, which were tied to attendance.

Our department's bonus is directly linked to workload and attendance days. If you take a month of sick leave, not only is that month's performance pay gone, but your year‐end bonus will be significantly affected. To put it bluntly, if you get sick, you bear the financial loss yourself. Who would dare to rest easily? (N9)

…It has a direct impact. The hospital has a clear rule that during sick leave, a corresponding amount of salary will be deducted based on the number of days off. If it's 15 days or more, you cannot participate in any year‐end awards or promotions… (N23)

This fear made them feel like ‘flawed assets,’ disadvantaged in their career progression, and engendered a sense of fear and insecurity.

After being injured, you become very sensitive. You wonder if your boss will think you're physically unfit and unreliable, so they won't consider you for learning or promotion opportunities. This worry stays with you. (N11)

#### 3.2.3 The Absence of Proactive and Ergonomic Support

The personal experience of WMSDs gave nurses a clearer and more critical perspective on the organization's long‐standing failure in injury prevention. They reflected on their work environment, pointing out severe deficiencies in the provision of safe patient handling equipment (e.g., patient lifts), effective ergonomics training, and the cultivation of a ‘safety‐first’ culture.

We chant ‘patient‐centered care’ every day, but who is ‘nurse‐centered’? The patient lift in our unit is either broken or too cumbersome to use, so everyone still resorts to manual handling. If the hospital really cared about us, it would provide the equipment and proper training, not just talk after we've all injured our backs. (N8)

From their viewpoint, the organization's failure to make a concerted effort to prevent foreseeable injuries was, in itself, the most fundamental form of neglect and lack of support.

There's training every year, but it's just a formality, all theoretical. No one ever comes to the clinical setting to teach us, hand‐in‐hand, how to move an agitated patient in a tiny space in the most labor‐saving and safest way. Our injuries, many of them, were avoidable. (N5)

### 3.3 Theme 3: Long‐Term Ramifications of Inadequate Support on Professional Identity and Career Trajectory

This theme explores the long‐term and often irreversible impact of WMSDs and the subsequent support experiences on nurses. It moves beyond the immediate consequences of the injury to delve into how this experience reshaped the nurses' relationship with their profession and organization, as well as their plans for the future.

#### 3.3.1 Erosion of Organizational Loyalty and Trust

Feeling abandoned by the organization during their most vulnerable moments fundamentally destroyed the psychological contract nurses had with the hospital. Many who had once considered the hospital their second home and felt a strong sense of collective pride began to see themselves as nothing more than replaceable cogs in a massive medical machine.

I used to think that if I worked hard for the hospital, the hospital would be my safety net. But this injury made me see clearly: when you fall, the organization won't truly feel sorry for you. It will just find someone to replace you. That sense of belonging is gone in an instant. (N2)

This feeling of being ‘instrumentalized’ led to emotional detachment and a sharp decline in organizational loyalty.

Trust is something that, once broken, is hard to piece back together. Now, I go to work, complete my tasks, and collect my salary. I no longer treat the department's and hospital's business as my own. My heart has grown cold. (N20)

#### 3.3.2 Re‐Evaluating the Future: The Genesis of Turnover Intentions

For many participants, the injury and the disappointing support that followed acted as a significant ‘catalyst,’ prompting them to seriously consider leaving their current position or even the nursing profession altogether.

After this back injury, I really started to seriously consider a career change. I'm only in my early 30s, and my back is already like this. What will I do in the future? I don't want to spend the rest of my life in pain and on medication. This profession… maybe it's just not for me anymore. (N12)

They began to question whether a career that demanded such a heavy physical and emotional toll, yet failed to provide adequate protection at critical moments, was worth a lifetime of dedication.

The doctor suggested I switch to a less demanding department or just leave clinical work entirely. The thought of quitting has crossed my mind. I feel like my body has been completely overdrawn, and the care and reward you get are too little in return. It's not proportional. (N4)

#### 3.3.3 A Weakened Professional Identity

The combination of physical limitations from the WMSD and the psychological burden of feeling like a ‘hindrance’ eroded the nurses' core professional identity as competent ‘caregivers.’

Sometimes even my walking posture changes. Colleagues would joke, ‘Why are you walking like an old lady?’ Though no harm was meant, the comment struck a chord. You become afraid—afraid that others will think you're no longer capable of doing your job. That fear stays with you. (N14)

They were caught in a profound cognitive dissonance: as professionals meant to care for others, they were now unable to care for themselves or fully perform their duties.

The worst part is that feeling of powerlessness. You see a patient who needs help, but your back won't let you exert force. You're a nurse, but you can't even perform the most basic nursing care. At that moment, you doubt your own value, you doubt if you can still be a good nurse. (N17)

This triggered doubts about their own capabilities and anxiety about their future professional performance.

To be honest, there was a moment after the injury when I did waver. But the sense of responsibility that comes with nursing made me want to focus on recovering first, so that I could eventually return to my post. (N22)

#### 3.3.4 The Burden of Self‐Advocacy

In the absence of a proactive and clear organizational support system, nurses found they had to become their own advocates. They had to actively fight for reasonable work adjustments, painstakingly explain the necessity of their rest to managers, and navigate complex administrative procedures alone. This process of self‐advocacy was in itself a tremendous drain on their emotional and mental energy, adding a heavy layer of psychological burden on top of their physical pain.

You have to ask about everything, fight for everything yourself. No one will come to you and tell you what rights you have or what support is available. You have to be a ‘troublemaker’ to negotiate with leaders and grind it out with HR. The process itself is utterly exhausting. (N24)

It feels like fighting a battle alone. On one hand, you're fighting with your own body; on the other, you're contending with this massive, indifferent system. It's just so tiring. (N13)

## 4 Discussion

This study provides a nuanced account of how Chinese nurses experience organizational support after WMSDs. The key finding is not simply that support was insufficient, but that support was fractured: nurses often received immediate relational help from managers and colleagues while encountering formal systems that felt bureaucratic, opaque, and weakly responsive to pain, recovery, and return‐to‐work needs. This distinction helps clarify why some nurses described moments of warmth within departments yet still interpreted the organization as failing to care for them.

Viewed through OST, this fractured support carries important theoretical meaning. Relational support from colleagues and nurse managers partially met nurses' socioemotional needs for empathy, respect, and belonging (Almalki et al. 2025; Mertens et al. 2025). However, informal support could not replace systematic organizational support, including fair procedures, safe working conditions, ergonomic resources, and transparent return‐to‐work pathways (Eisenberger et al. 2002; Rigi et al. 2025; Larsman et al. 2024). When formal responses emphasized staffing needs, performance‐pay losses, or rapid return to work, nurses interpreted these actions as signals that their contribution was valued mainly when they remained fully productive. This low perceived valuation weakened trust, affective commitment, and willingness to remain in the organization, consistent with the negative outcomes predicted by OST (Rhoades and Eisenberger 2002; Larsman et al. 2024; Muhaidat et al. 2025; Nurmeksela et al. 2025; Li et al. 2025).

The findings also extend international evidence on occupational injury and return to work. The central role of the manager, ambiguity of return‐to‐work arrangements, and injury‐related threats to professional identity have been reported across settings (Almalki et al. 2025; Baril et al. 2003; Jones, Juby, et al. 2023; Jones, O'Greysik, et al. 2023). In the Chinese nursing context, however, two features sharpened these dynamics. First, participants' desire not to burden colleagues intensified guilt and presenteeism, making informal collegial help both supportive and psychologically costly (Abou Hashish 2017; Zong et al. 2025; Zhang, Wang, et al. 2025). Second, persistent staffing pressure limited the feasibility of light‐duty work, longer sick leave, and phased return, creating direct tension between nurses' recovery needs and departmental operations (Dall'Ora et al. 2025; Baril et al. 2003; Li et al. 2021).

The findings have direct implications for nursing management, hospital administration, and health policy. Hospitals should move from reactive injury management to proactive investment in nurse well‐being (Esmaeili et al. 2025). Primary prevention should include safe patient‐handling equipment, ergonomic redesign of work processes, practical ward‐based training, and adequate staffing (Krishnanmoorthy et al. 2025; Abdul Halim et al. 2025; Scheer et al. 2025). After injury, standardized and phased return‐to‐work programmes should specify duties, duration, assessment criteria, and follow‐up so that injured nurses are not forced to negotiate support alone (Zhang, Du, et al. 2025). Performance‐pay policies should also protect nurses with confirmed WMSDs from disproportionate financial penalties; one option is a centralized occupational‐health compensation mechanism that prevents the burden from falling solely on individual nurses or their units (Muhaidat et al. 2025; Wu et al. 2025). Nurse leaders should be trained to identify physical and psychological distress, communicate with empathy, advocate for reasonable adjustments, and cultivate a safety‐first unit culture rather than normalizing working through pain (Zong et al. 2025; Fan et al. 2025; Zhang et al. 2023). At the policy level, national guidance on occupational injury management in healthcare institutions is needed to support consistent return‐to‐work standards and sustain the nursing workforce (Vallée 2024; Janes et al. 2025; Bailey et al. 2025; Woodnutt et al. 2025).

This study has some limitations. First, the sample was primarily drawn from several large tertiary hospitals in a specific geographical region, which may limit the generalizability of the findings to other regions or to smaller, primary care facilities. Second, the study relied on retrospective interviews, and participants' recall of their post‐injury feelings may be subject to bias. Finally, this study did not include the perspectives of hospital administrators or human resources personnel; future research could employ a multi‐stakeholder approach to gain a more comprehensive understanding of this issue.

## 5 Conclusions

In conclusion, this study reveals that organizational support for Chinese nurses suffering from WMSDs is fragmented, reactive, and insufficient. Its contribution is to show how the mismatch between informal relational support and weak formal systems shapes nurses' recovery experiences, perceived professional value, and retention intentions. For nursing practice and science, the findings highlight the need to treat post‐injury support as a workforce‐sustainability issue rather than a purely administrative matter. Structured return‐to‐work pathways, ergonomic prevention, fair performance policies, and stronger nurse‐leader advocacy are essential for safeguarding nurses' health and maintaining resilient patient‐care systems.

## Author Contributions

**Kejimu Sunzi:** conceptualization, methodology, investigation, formal analysis, writing – original draft, software. **Yihong Lin:** conceptualization, investigation, data curation, resources, writing – original draft. **Li Guo:** data curation, investigation, resources, writing – original draft. **Fan Li:** writing – original draft, investigation, data curation, resources. **Cheng Lei:** funding acquisition, project administration, supervision, writing – review and editing, methodology, conceptualization. **Wen Zhou:** data curation, supervision, writing – review and editing, methodology. **Zhiyun Yang:** investigation, writing – original draft, data curation, resources. **Qi Huang:** conceptualization, investigation, formal analysis, validation, writing – original draft.

## Funding

This work was supported by Chongqing Higher Education Teaching Reform Research Project (252013) and Southwest Medical University Higher Education Teaching Research and Reform Project (JG2023jdyb052).

## Ethics Statement

The study was approved by the ethics committee of Deyang People's Hospital (Approval No. 2025‐04‐043‐K01). Written informed consent was obtained from all participants before audio recording began.

## Conflicts of Interest

The authors declare no conflicts of interest.

## Supporting information


**Supporting Information: S1** Semi‐Structured Interview Guide.

## Data Availability

The data that support the findings of this study are available on request from the corresponding author. The data are not publicly available due to privacy or ethical restrictions.
