# Supplementary material for: The Experiences and Perceptions of Organizational Support Among Chinese Nurses Following Work‐Related Musculoskeletal Disorders: A Qualitative Descriptive Study
Source: Nurs Open. 2026 Sep 7;13(9):e70793. doi: 10.1002/nop2.70793 (PMC13548136; doi:10.1002/nop2.70793)
Supplement: Supplementary file 1 [file NOP2-13-e70793-s001.docx]

Supplementary S1. Semi-Structured Interview Guide.

**Research Project: A Qualitative Study of Organizational Support Experiences of Chinese Nurses with Work-related musculoskeletal disorders**

**Interviewer:** ___________

**Participant Code:** ___________	**Date:** ___________

**Gender:** ___________	**Age:** ___________ **Education:** ___________

**Marital Status:** ___________ **Department:** ___________ **Years of Work:** ________

#### **I. Opening and Informed Consent**

"Hello. Thank you very much for taking the time to participate in this interview. My name is [Interviewer's Name], and I am a researcher from[Research institution's Name].

The purpose of this study is to gain an in-depth understanding of the real experiences and feelings of nurses regarding organizational support after experiencing work-related low back pain or injury. By listening to your story, we hope to provide valuable insights that can help improve the occupational health environment for nurses.

Today's interview will take approximately 30-60 minutes. This will be more of a relaxed conversation; there are no right or wrong answers. We are genuinely interested in your personal experiences and perspectives. With your permission, I will be audio-recording our conversation. This is purely to ensure I can accurately capture what you share. All information will be kept strictly confidential, and your identity will be anonymized in any research reports. You may ask to pause or stop the interview at any time.

Is everything clear so far? Do you agree to begin and for me to start the recording?"

*(Confirm consent and begin recording.)*

#### **II. Background Information (Building Rapport)**

*Purpose: To establish a comfortable atmosphere and understand the participant's professional background.*

1. Could we start by talking about your nursing career? How many years have you been a nurse?
2. What is your current department? How long have you been working there?
3. Overall, would you say your daily work involves a heavy physical workload?

#### **III. The Low Back Pain (LBP) Incident**

*Purpose: To contextualize the injury event, which will serve as the basis for discussing the organizational response.*

1. Can you recall and describe in detail the most recent and memorable experience you had with work-related low back pain or injury?
   - **Probes:** What specific task were you performing at that moment? (e.g., lifting a patient, bending over for a long time)
   - **Probes:** What was your immediate feeling when it happened?

#### **IV. Injury Reporting Process and Initial Organizational Response**

*Purpose: To explore the functioning of the formal support system and the nurse's perception of procedural fairness.*

1. What was the first thing you did after the injury? Who did you report it to?
2. Can you describe the entire process of reporting this injury?
   - **Probes:** Did you find the reporting procedure to be clear and easy to follow?
   - **Probes:** What was your impression of the administrative staff you dealt with (e.g., HR, occupational health)? What were their main concerns?
   - **Probes:** Did you feel that your injury was taken seriously? Why or why not?

#### **V. Interactions with Managers and Colleagues**

*Purpose: To investigate the informal support system (especially from direct supervisors and peers), which is a key source of perceived organizational support.*

1. How did your head nurse (or direct manager) find out about your injury? What was her/his initial reaction?
   - **Probes:** During your recovery, what specific help or arrangements did your head nurse provide? (e.g., adjusting your schedule, reducing your workload)
   - **Probes:** How would you describe your head nurse's role and attitude throughout this entire event? How did that make you feel?
2. How did your colleagues react to your injury?
   - **Probes:** Did they help share your workload or offer other forms of assistance?
   - **Probes:** When your colleagues helped you, what were your feelings? (e.g., grateful, guilty, pressured)

#### **VI. Sick Leave and Return-to-Work Experience**

*Purpose: To assess the organization's concrete support measures regarding employee well-being and working conditions.*

1. How long did you take off work for this back injury? Was the process of applying for sick leave smooth?
2. During your leave, did the hospital or your department contact you? What was that contact like?
3. When you were preparing to return, did the hospital or department create a return-to-work plan for you?
   - **Probes:** Can you describe what it was like when you first returned to your post? Were your job duties and intensity adjusted?
   - **Probes:** Did you feel these adjustments were adequate? Were they based on your physical condition or the department's needs?
   - **Probes:** How long did this "light-duty" or modified role last? Was there clear communication about when you would resume your normal duties?

#### **VII. Overall Perception of Organizational Care and Long-Term Impact**

*Purpose: To comprehensively assess the participant's overall Perceived Organizational Support (POS) and explore its subsequent impact on work attitudes and career planning,.*

1. Looking back on the entire experience, from the injury to your recovery and return to work, to what extent did you feel that your hospital (as an organization) cared about your well-being and valued your contributions?
   - **Probes:** Can you give any specific examples that made you feel "the organization cares about me"?
   - **Probes:** Were there also moments that made you feel "the organization doesn't really care"?
2. Do you believe this injury and the subsequent experience had any impact on your financial income, performance reviews, or future promotion opportunities? Why do you think so?
3. Has this experience changed your view of the nursing profession or of the hospital where you work?
   - **Probes:** Has it affected your passion for your work or your sense of belonging to the hospital?
   - **Probes:** Have you considered changing departments or even leaving the profession as a result?
4. If you had the opportunity to give advice to the hospital management, what would be the most important thing you would say to better support nurses who get injured like you did?

#### **VIII. Closing the Interview**

"That's about all the questions I have for today. Looking back at our conversation, is there anything else you feel is important that we haven't touched upon, or anything you'd like to add?

Thank you again for your candid sharing. Your experience is incredibly valuable to our research. In the future, as I analyze the data, would it be okay if I contacted you again via to clarify any information? Thank you so much!"

*(Confirm and stop recording.)*
